# Mapping the landscape of congenital anomaly registries worldwide: what role do health and economic factors play?

**DOI:** 10.7189/jogh.16.04263

**Published:** 2026-08-17

**Authors:** Martina Schatz, Felicity Vidya Mehendale

**Affiliations:** 1 Medical School, University of Edinburgh, Edinburgh, Scotland, UK; 2 Centre for Global Health Research, Usher Institute, University of Edinburgh, Edinburgh, Scotland, UK

**Keywords:** congenital anomaly surveillance, population health data, health inequality, global health, neonatal and perinatal outcomes

## Abstract

**Background:**

Congenital anomalies (CAs) are structural or functional changes occurring during intrauterine life. The World Health Organization data indicate that about 295,000 babies die within the first four weeks of birth due to CAs every year. It has been estimated that approximately 94% of severe CAs occur in low- and middle-income and low-income countries. While the use of a national congenital anomaly registry (CAR) can help improve healthcare quality, access, and planning for families and children born with CAs, few countries report using such a system. We aimed to evaluate the correlation of health and economic indicators with the existence of national CARs.

**Methods:**

We compiled data on individual countries’ reported CAR status from regional networks, the International Clearinghouse for Birth Defects Surveillance and Research, the Modell database, and a literature search. We classified countries according to the reported CAR status into those that reported having a national CAR with or without regional CAR, only a regional CAR, and no CAR data available. Using the Kruskal–Wallis test, we analysed whether a country’s CAR status was correlated with health and economic data from UNICEF and the World Bank.

**Results:**

Of the 202 countries in our sample, 30 reported having a national CAR, 19 only a regional CAR, and 153 no CAR. Country income group-level analysis showed that 35.4% of high-income, 13.2% of upper middle-income, and 0% of low- and middle-income, and 0% low-income countries were reported to have national CARs. Countries lacking a national CAR tended to have a lower gross domestic product (*P* < 0.001), increased infant mortality rates (*P* < 0.001), and lower birth registration rates (*P* < 0.001).

**Conclusions:**

While the importance of CARs for improving global health is widely recognised, most LMICs and LICs still do not report having a national CAR. Our study suggests that countries without CARs face greater economic and health systems challenges. Collaborations to develop national CARs need to consider the impact of these wider economic and health systems challenges.

Congenital anomalies (CAs) are structural or functional changes that occur during intrauterine life. According to the World Health Organization (WHO), about 295,000 babies die within the first four weeks of birth due to CAs every year. Additionally, CAs rank 17th among the causes of the global burden of disease [1]. Estimates indicate that 94% of severe CAs occur in low- and middle-income countries (LMICs), and that many of these countries lack rigorous surveillance programmes, making accurate calculations of incidence and prevalence more difficult [2,3]. Understanding the epidemiological profile of CA through national-level monitoring and the analysis of data obtained through this monitoring provides countries with an opportunity to evaluate its impact on the population. This also generates useful data that can inform health policy to ensure equitable access to timely and high-quality care, thus reducing morbidity and mortality [4].

While the United Nations Children’s Fund (UNICEF) and other organisations collect data on various health and economic indicators, and while Modell and colleagues developed a dataset to estimate the global burden of CAs [5], there is no global database documenting the existence of national congenital anomaly registries (CARs) for individual countries. There is a need to curate data from each of these databases to map a global picture of prevalence of national CARs [5]. This lack of national CARs may be the result of a wide range of economic, health, policy, infrastructural, and governance barriers. Unlike other registries (*e.g.* cancer registries or registries for other acquired adult diseases), the establishment of CARs is further complicated by the high proportion of unattended births and inadequacies in civil birth registration systems, in many countries, globally [6].

Article 7.1 of the United Nations Convention on the Rights of the Child states that all children should be registered immediately after birth, have the right to a name and nationality, and have the right to know and be cared for by their parents [7]. However, the UNICEF reports that 4 in 10 children aged <5 years, amounting to around 166 million worldwide, are living without being registered, with most being from Asia and sub-Saharan Africa. This means that the children’s existence or legal proof of their identity are never formally established [8]. Factors associated with such marked global disparities in achieving basic birth registration for every child, as well as economic disparities are also likely to present barriers to CAR development. However, the association between these factors and the presence or absence of a national CAR has not been reported.

We therefore aimed to determine which countries are reported in existing global databases to have national and/or regional (subnational) CARs, and to evaluate the correlation between the existence of a national and/or regional CAR and universally-used health indicators (birth registration rates (BRR) and infant mortality rates (IMRs)) and economic indicators (World Bank (WB) country income group, gross domestic product (GDP), and health expenditure).

## METHODS

Data on the existence of reported CARs in all UNICEF listed countries were retrieved and correlated with health and economic indicators from UNICEF [9] and the WB [10].

### CARs

Based on the UNICEF list of 202 countries, we searched for documentation of the existence of national or regional (subnational) reported CARs [9]. Data regarding the existence of reported national or regional CARs worldwide were collected by cross-referencing standalone databases or clearing houses to which individual countries in specific global regions submit data about their CARs. Since data on countries’ reported CAR status had to be curated from multiple sources, we reported our retrieval and representation of data per the GATHER checklist [11].

We identified the main organisations that host data on countries’ CARs from the list of surveillance networks on The Global Health Network [12]. Others were identified though online searching and cross-referencing links from published reports from each relevant organisation. Others were identified though online searching and cross-referencing links from peer-reviewed literature and from published reports from each relevant organisation. Our aim was to find any reference to the existence of a national CAR irrespective of time period.

We identified the following international organisations: Modell’s Global Data set of Congenital Disorders (MGDb) [5], the International Clearinghouse for Birth Defects Surveillance and Research (ICBDSR) [13], the European Surveillance of Congenital Anomalies (EUROCAT) [14], the Red *Latinomericana de Malformaciones Congenitas* (Re*LAMC*) [15], South-East Asia Regional – Newborn and Birth Defects Database (SEAR-NBBD) [16] and sub-Saharan Congenital Anomaly Network (sSCAN) [17]. Single condition registries (national or regional) were not included in this study.

The MGDb data set was developed as a mechanism of estimating prevalence and effects of interventions on mortality and disability due to CA, and therefore provides a framework for objective assessment of their burden [18]. We used this database as a useful resource to crosscheck whether we were missing important data, as it is the only evidence-based report that contains this data on a global scale. The EUROCAT and Re*LAMC* are networks of registries for the epidemiological surveillance of CAs in Europe and South America, respectively, which provide similar, yet incomplete data on the national and regional percentage CAR coverage. The ICBDSR, which provides a similar dataset, aims to ‘conduct worldwide surveillance and research to prevent birth defects’ [13]. The SEAR-NBBD and sSCAN had no official registers, but stated they are developing CARs for the relevant regions. We additionally cross- referenced published review articles on CARs, and identified one review [4] that listed CARs that were not listed in other databases.

Regional CARs represented subnational data, although was it not always clear which exact geographic area is covered. They were included in our data because they were incorporated in the databases we used, and because it would be inappropriate to place these countries in the same category as countries with no CAR at any level.

We did not include CARs for single congenital conditions or anatomically discrete regions, since their presence cannot necessarily serve as a proxy for whether a country has a national CA surveillance programme. Importantly, the comprehensiveness of reported CARs in terms of the range of conditions included was not specified consistently; therefore, it possible that current CARs may not include all major and minor congenital anomalies.

Given that factors such as whether a CAR is hospital- or population-based, or the number of conditions covered by a CAR, we deemed any country reported as having such a CAR in at least one of the above sources as having a national CAR with or without regional CARs; having only a regional (subnational) CAR; and having no CAR data available (Table S1 in the **Online Supplementary Document**).

### UNICEF and World Bank data

We retrieved BRR data for children aged <1 and <5 years as a .csv file from the annually-updated UNICEF dataset up to August 2024 [9]. These data were particularly relevant for our analysis, considering that CAs should be registered at or soon after birth. We further selected three relevant population health and economic indicators: the average GDP per capita (in USD), average health expenditure (% of GDP), and IMR per 1000 births. We selected these parameters because they are widely used indicators of both economic and health metrics, and because most countries had their data readily available on the WHO and WB sites. The most recent data for the first two indicators were from 2023; however, IMR data for 2023 were incomplete, so we used the 2022 dataset for analysis. We also classified countries by region and income according to the WB country classification. We excluded Venezuela from the analysis of countries by income, as it is currently ‘unclassified’ by the WB [10]. We did not include the Gini index because the global data for this indicator were incomplete and were unavailable for many countries.

### Merging of datasets and analysis

We imported the data from .csv files into Microsoft Excel, version 16.109.3 (Microsoft, Redmond, Washington, USA) for initial screening and merging of data sets. There were differences in the list of countries obtained from the UNICEF and the WB. Specifically, the WB dataset included 217 countries, of which 21 were absent from UNICEF dataset, while the UNICEF dataset included 202 countries, with six being absent from the WB dataset (Anguilla, Cook Islands, Holy See, Montserrat, Niue, Tokelau). Therefore, we included all 202 countries listed by the UNICEF to determine the total number of countries with a CAR. For all analyses based on indicators from both the WB and UNICEF, we included only the 195 countries.

We used the Kruskal–Wallis test was used to test for statistical significance between health and economic indicators against the three categories of registry status (Table S2 in the **Online Supplementary Document**). All data analyses were performed on R, version 4.3.1 (R Core Team, Vienna, Austria).

## RESULTS

**Figure 1:**
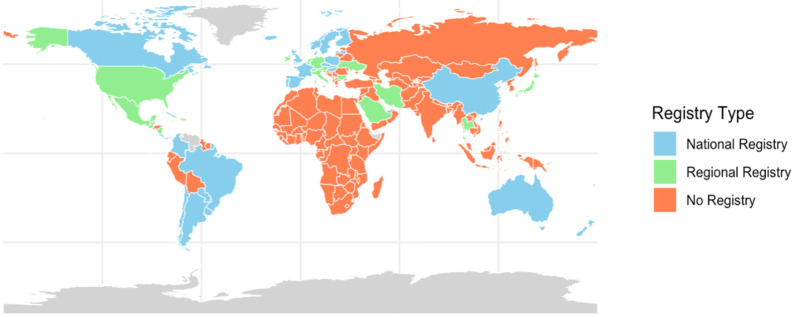
Map of the world displaying 202 countries and their CAR status as recorded in data sources listed in the methods section. Blue box displays countries with national CARs (n = 30, 14.9%), green box displays countries with regional CARs only (n = 19, 9.4%), and orange box displays countries with no registries at all (n = 153, 75.7%).

The analysed reports indicate that, of 202 countries, 153 (75.7%) had no CAR, only 30 (14.9%) countries had a national CAR, and 19 (9.4%) had a regional (subnational) CAR, with significant differences across continents (**Figure 1**). However, data on how many regions of a country or what percentage of population were not consistently available, making it likely that these regional CARs exist only in selected regions of individual countries.

### Correlation with economic and health indicators

Six countries from the UNICEF dataset had no CARs, and one more country (Venezuela) had no data in the World Bank dataset. This left 195 countries for the correlation analysis.

Countries lacking a national CAR tended to have lower GDPs, increased IMRs and lower BRRs. There was a strong correlation between a higher income status and the likelihood of having a national CAR. Analysis by country income group showed that national CARs existed for 35.4% of high-income countries (HICs), 13.2% of upper middle-income countries (UMICs), 0% of LMICs, and 0% of low-income countries (LICs) (**Table 1**).

**Table 1:** Number of countries by income classification and relevant prevalence of CARs

Income group	Number (%) of countries (n = 195)*	National CAR (n = 30)*	Regional CAR (n = 19)	No CAR (n = 146)
HIC	65 (33.3)	23 (35.4)	12 (18.5)	30 (46.2)
UMIC	53 (27.2)	7 (13.2)	6 (11.3)	40 (75.5)
LMIC	51 (26.2)	0 (0.0)	1 (2.0)	50 (98.0)
LIC	26 (13.3)	0 (0.0)	0 (0.0)	26 (100.0)

CAR – congenital anomaly registry, HIC – high-income country, LIC – low-income country, LMIC – low- and middle-income country, UMIC – upper middle-income country

*There were 195 countries in total because data was missing for Venezuela.

Higher income was related with having a higher BRR for children aged <1 and <5 years. In all country income groups, the CARs for children aged <1 year had fewer data available compared to those for children aged <5 years. Out of 195 countries, 84 (43.1%) lacked a CAR for children aged <1 year, compared to 15 (7.7%) lacking a register for children aged <5 years. Additionally, LICs had a BRR of only 53.7% for children aged <1 year (**Table 2****)**.

**Table 2:** Relationship between income group and average GDP *per capita* in current USD, average health expenditure as % of GDP, average IMR per 1000 births, and average BR*

Income group	Average GDP *per capita*	Average health expenditure	Average IMR	Average BRR <1	Average BRR <5
HIC	44,968.9	8.1	5.3	93.5	99.7
UMIC	8,558.2	6.8	14.7	91.3	94.5
LMIC	2,571.6	5.6	27.6	70.0	77.8
LIC	792.6	6.6	43.2	53.7	57.9

BRR – birth registration rate, CAR – congenital anomaly registry, HIC – high-income country, IMR – infant mortality rate LIC – low-income country, LMIC – low- and middle-income country, UMIC – upper middle-income country

*We additionally correlated the data to birth registration rates in under 1 years and under 5 years from the UNICEF data set.

Despite wide variation in GDP between different income groups (GDP *per capita* ranged from USD 793 to USD 44,969), the percentage of healthcare expenditure varied less significantly across countries (**Table 2**). Specifically, LMICs spent the least on healthcare (5.6%), followed by LICs (6.6%), and UMICs (6.8%), while HICs spent 8.1% (only 1.5% more than LICs). We found no correlation between countries’ CAR status and percentage of healthcare expenditure. Countries with no registry had the lowest GDPs *per capita* and percentages of healthcare expenditure, as well as lowest BRRs and highest IMRs (**Table 3**).

**Table 3:** Relationship between CARs status and average GDP *per capita* in current USD, average health expenditure as % of GDP, average IMR per 1000 births, and average BR*

Countries’ CAR category	Average GDP *per capita*	Average health expenditure	Average IMR	Average BRR <1	Average BRR <5
National CAR	37,543.28	8.79	5.04	88.52	98.44
Regional CAR only	38,887.59	8.69	7.12	93.14	98.30
No CAR	11,326.25	6.27	23.18	72.91	81.92

BRR – birth registration rate, CAR – congenital anomaly registry, GDP – gross domestic product

*We additionally correlated the data to birth registration rates in under 1 years and under 5 years from the UNICEF data set.

**Figure 2:**
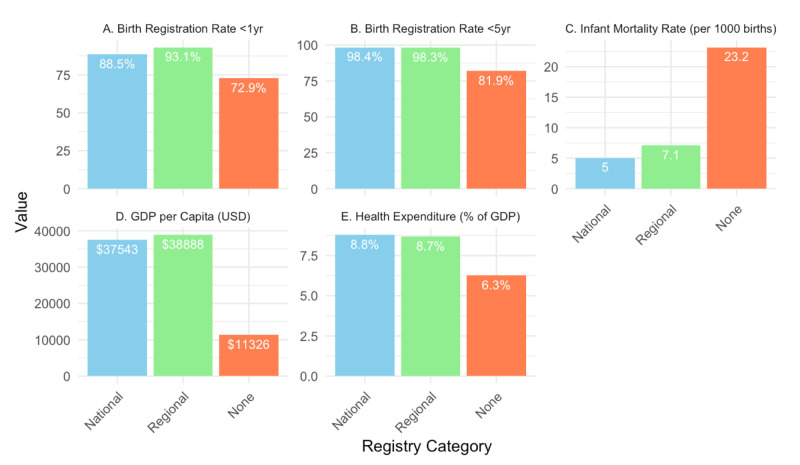
Bar charts displaying relationships of three CAR statuses with a birth registration rate <1 year (**Panel A**), a birth registration rate <5 years (**Panel B**), IMR per 1000 births (**Panel C**), GDP *per capita* in current USD (**Panel D**), and health expenditure as % of GDP (**Panel E**).

**Figure 3:**
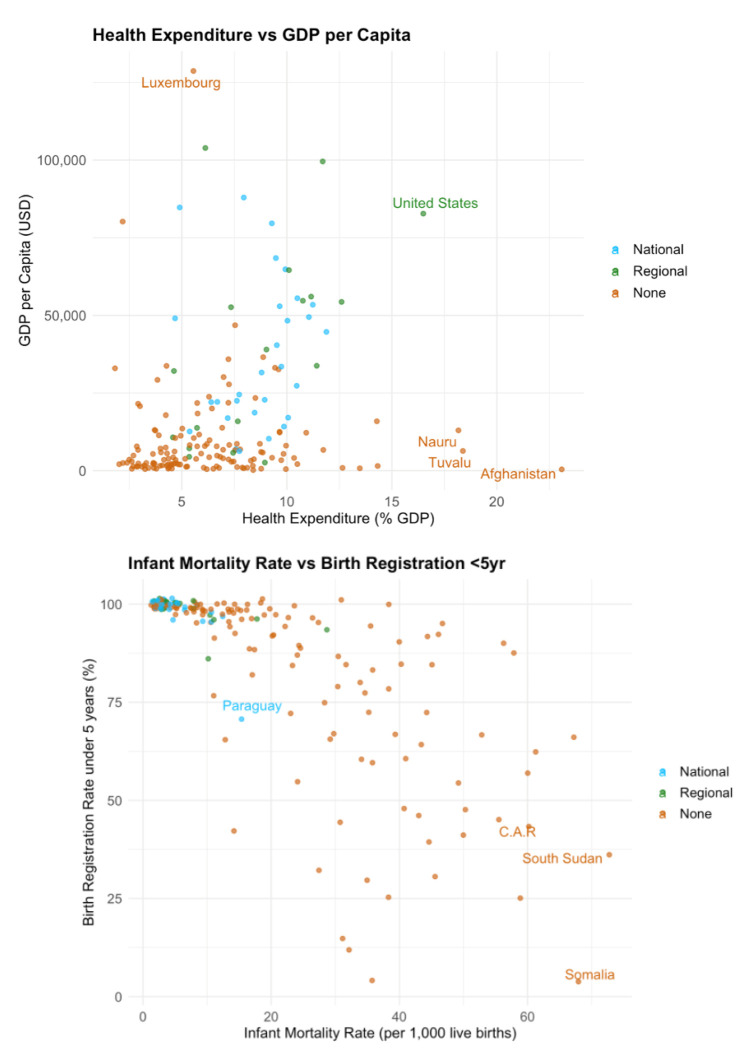
Scatter plots comparing characteristics of countries by CAR status based on economic (**Panel A**) and health indicators (**Panel B**). Monaco, with a GDP 256 580, is not displayed in the graph. C.A.R. – Central African Republic.

We found no significant difference between the national and regional CARs data (**Figure 2**), although when compared to the group of countries with no CAR, all correlations were significant, except for BRR <1 year of age (*P* < 0.001). We created scatter plots to study the economic indicators GDP per capita and health expenditure, with each country represented by a single dot (**Figure 3**, Panel A). It was important to separate each country as a singular entity before grouping all LICs into one category with a specific outcome. We also assessed the correlation between registry status and BRR and IMR (**Figure 3**, Panel B).

### Economic indicators

Countries with lower health expenditure and GDP *per capita* were more likely to have no CAR (**Figure 3**, Panel A). We found mixed results for HICs, with some having national CARs and other with only regional CARs.

Countries with the highest GDP, namely Monaco and Luxembourg, did not have a CAR. After excluding Monaco due to being an outlier with a GDP of 256,580, we noted a much wider variation in GDP than in healthcare expenditure, with several low GDP countries spending a greater percentage on healthcare. For example, Afghanistan spent approximately 23% on healthcare, although it had a GDP *per capita* of 415 and no CAR. The USA was the only country with any form of CAR that spent more than 15% on healthcare.

### Health indicators

As we did not have sufficient data for BRRs <1 years of age, we chose to focus on data for those aged <5 years. Paraguay was only country with a CAR that had a BRR of <75% for those aged <5 years (**Figure 3**, Panel B). All other countries below this threshold did not have a CAR. Countries with national and regional registries tended to have lower IMR and higher BRR. There was a larger spread between countries with no registry, with some such as Central African Republic, Sudan and Somalia having very low BRR (<50%) and high IMR (>60/1000).

## DISCUSSION

### Burden of disease of CAs and the absence of registries

The value of CARs is well recognised, with a global consensus reached that they could improve health outcomes for general populations, as well as healthcare access and planning [4]. By registering children born with CA, we can create databases with essential epidemiological information that could inform preventive screening and healthcare service planning and allow for the identification of risk factors for CAs [4]. Despite this, we found that only globally 14.9% of countries have national CARs, and that there are significant differences in key health and economic indicators between these countries and those without a CARs.

Every LIC included in the study and most LMICs (98%) had no CAR, potentially leading to an underrepresentation of the prevalence of CAs in low-income settings [2]. In contrast, most children in HICs are born with immediate contact to healthcare professionals that diagnose, report, and manage CAs, leading to overestimates of the proportion of CAs in such contexts. All these factors may impact the accuracy of public health programmes and lead to underestimates in population statistics in LICs [19].

### The role of birth registration

We found major variation in BRR among countries with no CAR, with 14.5% having a BRR of <50% even by the aged <5 years, which presents major challenges to CAR development. Children who are not registered are more likely to miss out on public services that help secure their fundamental rights, such as healthcare and education, and are in greater danger of exploitation [20]. Unless the process of birth registration is accelerated, the total number of unregistered children in sub-Saharan Africa will continue to increase and will exceed 100 million by 2030 [4]. This absence of reliable data for births makes a large proportion of the world’s population unseen and unaccounted for and has been described as ‘a scandal of invisibility’ [21].

Similarly, we found an IMR of 23.2 per 1000 births in countries with no CAR, in contrast to the significantly lower IMR of 5 per 1000 births among countries with a national CAR. Otherwise, the IMR was much higher in LICs, which tended to have no CAR. The Dominican Republic was the only country that had an IMR >20 per 1000 births and a regional CAR. Data from the WHO indicate that sub-Saharan Africa accounted for 57% of total deaths among children <5 years of age in 2022, with one of the most common causes being CAs [22]. Additionally, many of the neonatal deaths in this region were associated with lack of quality care at birth or access to treatment in the first days of life [21]. These drastic values emphasise the importance of attempting to create CARs to work towards Sustainable Development Goal (SDG) 3, which focuses on good health, and particularly SDG 3.2, which places child survival as a priority [23].

### Economic factors

Overall, 75.7% of countries, including some HICs, had no national CAR. Despite this, GDP emerged as a significant predictor of having a CAR, meaning that higher GDP countries were more likely to have a national CAR than LMICs (*P* < 0.001). Conversely, we noted that many LICs spent similar percentage of their GDP on healthcare as HICs, and that health expenditure alone did not correlate with CAR status, in contrast to our findings for GDP. These findings stress that the creation and maintenance of CARs is a multisystem challenge, and that solutions are needed that are potentially focused on infrastructure and systems as much as they are on pure health expenditure.

### Cultural and political contexts

In certain countries, cultural beliefs around CAs lead to stigmatisation, with mothers often being blamed for the malformation, consequently negatively impacting the disclosure of such pregnancies and registration of births [19]. Studies from rural India and Nigeria identified that parents of disabled children were socially marginalised because of beliefs that they had transgressed cultural taboos [24,25]. Children with CA are considered a burden to their families and communities and may even end up being neglected [19]. The examples given above are illustrative, rather than representative, as cultural factors are complex and stigmatising beliefs exist in all societies, including HICs. However, the higher proportion of births that are attended by skilled health professionals in HICs presents less of a barrier to early diagnosis, registration, and parental support [26].

Contrastingly, Finland hosts one of the most effective systems for CA surveillance and birth registration. Its CAR was established on 29 December 1962, while the registration of CA cases began on 1 January 1963. It contains national-level data on chromosomal and structural abnormalities; around 5000 new cases are reported annually, of which more than 2000 are major CAs. Finland’s CAR collects data on the current pregnancy, both maternal and foetal information, whether there are CAs in family members, and how the notification of the anomaly reached the family. Additionally, data are sourced from healthcare authorities and institutions, cytogenetic laboratories, Statistics Finland, National Supervisory Authority for Welfare and Health (*Valvira*), and various registers such as the Medical Birth Register, Register of Induced Abortions, and Care Register for Health. Finland’s CAR could be used as an exemplar when creating new registers and as a guide for improvement in current existing ones [27].

There is a strong need for a more standardised approach to the creation of these CARs, achieved through the sharing good practice and transferrable lessons. Clarity on the population covered in a country and specifying the types of CAs registered is required. Importantly, CARs serve the population and are not simply a data gathering exercise for epidemiological studies. The Re*LAMC*, for example, has been instrumental in supporting development in South America; as seen in Paraguay which has a achieved the national registry even though it has low BRR, potentially showing the value of regional networks [15]. Other regional networks of countries, such as sSCAN and SEAR-NBBD, aim to support a similar development [16,17]. While they have existed for a relatively short time, their impact could still be worth studying.

Our study has some limitations. We manually aggregated from various sources, increasing the risk of misclassification bias. The UNICEF and WB defined certain territories differently, thus leading to the exclusion of six UNICEF countries. Additionally, Venezuela was excluded because of no WB classification. Thus, we were only able to study correlation for 195 countries. We did not find any single source with details of national/regional CARs and the data they collect. Data on the presence of absence of a national or regional CAR were obtained from multiple sources. Although we cross-checked multiple sources to verify the existence of national or regional CARs, it is possible that a country has a national or regional CAR that is not currently listed in any of the sources we studied. This underlines the need for a reliable, robust and current registry of CARs. Additionally, we could not access individual CARs to obtain details of the number and type of CAs registered, or the percentage of the population covered. Thus, we studied only the reported existence of a CAR and not its quality. Data on regional CARs was likewise variable in completeness, yet we thought it important to assign countries with only a regional CAR to a separate category and not included them among countries with no CAR. To this, we note that regional CAR data are reflective only of specific regions and may not be representative of the entire country. This study identifies an association between variables; however, causation cannot be inferred. Importantly, it was not always clear if CARs were hospital- or population-based. Furthermore, registers tend to be transformative and may change over time, meaning that current data may only be accurate for the time when they were analysed.

## CONCLUSIONS

Most LMICs and LICs still do not have national CARs, despite their widely recognised importance for improving global health. We found that 75.7% of countries overall had no CAR at all and that only 14.9% had a national CAR. Our findings suggest that countries without CARs have broader, more complex health systems challenges, as reflected by their lower BRRs and higher IMRs. Countries with no registries had a BRR of 81.92% for children younger than 5 years of age and an IMR of 23.18%. Crucially, the most striking pattern was the impact of country income status, irrespective of whether countries spent a similar % of their GDP on healthcare. Importantly, the existence of these CARs should not be examined in isolation without considering the impact of broader economic and health systems challenges in the development and maintenance of CARs. This is reflected by SDG 16.9, which emphasises the importance of registration and the establishment of a legal identity for all newborns, which is, in turn, vital if we are to achieve SDG 3, good health and well-being, by 2030. Further collaborative work is needed to develop standardised approach to establishing CARs to create global datasets that can be used to further improve both birth registration and CA registration.

## Additional material


Online Supplementary Document

## Data Availability

**Data availability:** The curated dataset will be stored on the University of Edinburgh’ Datastore platform. Additional data are downloadable from UNICEF, World Bank, and WHO.
