# Supplementary material for: Mapping the landscape of congenital anomaly registries worldwide: what role do health and economic factors play?
Source: J Glob Health. 2026 Aug 17;16:04263. doi: 10.7189/jogh.16.04263 (PMC13480458; doi:10.7189/jogh.16.04263)

Supplement to: Schatz M, Mehendale F. Mapping the landscape of congenital anomaly registries worldwide: what role do health and economic factors play? J Glob Health. 2026;16:04263.

Table S1. Countries with registry presence and source

Countries	National CAR	Regional CAR	Source
Argentina	1	0	ReLAMC, ICBDSR
Australia	1	0	Cardoso-dos-Santos et al
Austria	0	1	EUROCAT
Belgium	0	1	EUROCAT
Brazil	1	1	ReLAMC
Bulgaria	0	1	EUROCAT
Canada	1	1	ICBDSR
Chile	1	1	ReLAMC, ICBDSR
China	1	1	Cardoso-dos-Santos et al
Colombia	1	1	ReLAMC, ICBDSR
Costa Rica	1	0	ReLAMC, ICBDSR
Cuba	1	1	ReLAMC
Czechia	1	0	EUROCAT, ICBDSR
Denmark	1	1	EUROCAT
Dominican Republic	0	1	Cardoso-dos-Santos et al
Finland	1	1	IEUROCAT, CBDSR
France	1	1	EUROCAT, ICBDSR
Germany	0	1	ICBDSR
Guatemala	0	1	Santos et al
Hungary	1	0	EUROCAT
Iceland	1	0	Santos et al
Iran (Islamic Republic of)	0	1	ICBDSR
Ireland	0	1	EUROCAT
Israel	0	1	ICBDSR
Italy	0	1	ICBDSR
Japan	0	1	Cardoso-dos-Santos et al, ICBDSR
Latvia	1	0	EUROCAT
Malta	1	0	ICBDSR
Mexico	0	1	ReLAMC, ICBDSR
Netherlands (Kingdom of the)	0	1	EUROCAT, ICBDSR
New Zealand	1	0	EUROCAT, ICBDSR
Nicaragua	0	1	ReLAMC
Norway	1	0	EUROCAT
Panama	1	0	Cardoso-dos-Santos et al, ReLAMC
Paraguay	1	1	ReLAMC
Poland	1	1	EUROCAT
Portugal	1	0	EUROCAT
Saudi Arabia	0	1	EUROCAT, ICBDSR

Singapore	1	0	Cardoso-dos-Santos et al
Slovakia	1	0	EUROCAT, ICBDSR
Spain	1	1	EUROCAT
Sweden	1	0	ICBDSR
Switzerland	0	1	EUROCAT
Thailand	0	1	Cardoso-dos-Santos et al
Ukraine	0	1	ICBDSR
United Arab Emirates	1	0	Cardoso-dos-Santos et al
United Kingdom	1	1	EUROCAT, ICBDSR
United States	0	1	ICBDSR
Uruguay	1	0	ReLAMC

Table outlining countries with national or regional (subnational) CARs and where the data was sourced from. Number 1 outlines presence of registry and 0 outlines the absence of a registry. Countries that are not in this table did not have any register in any of the sources used in the study.

Cardoso-dos-Santos et al (1)

Figure S1. PRISMA flowchart displaying country selection.

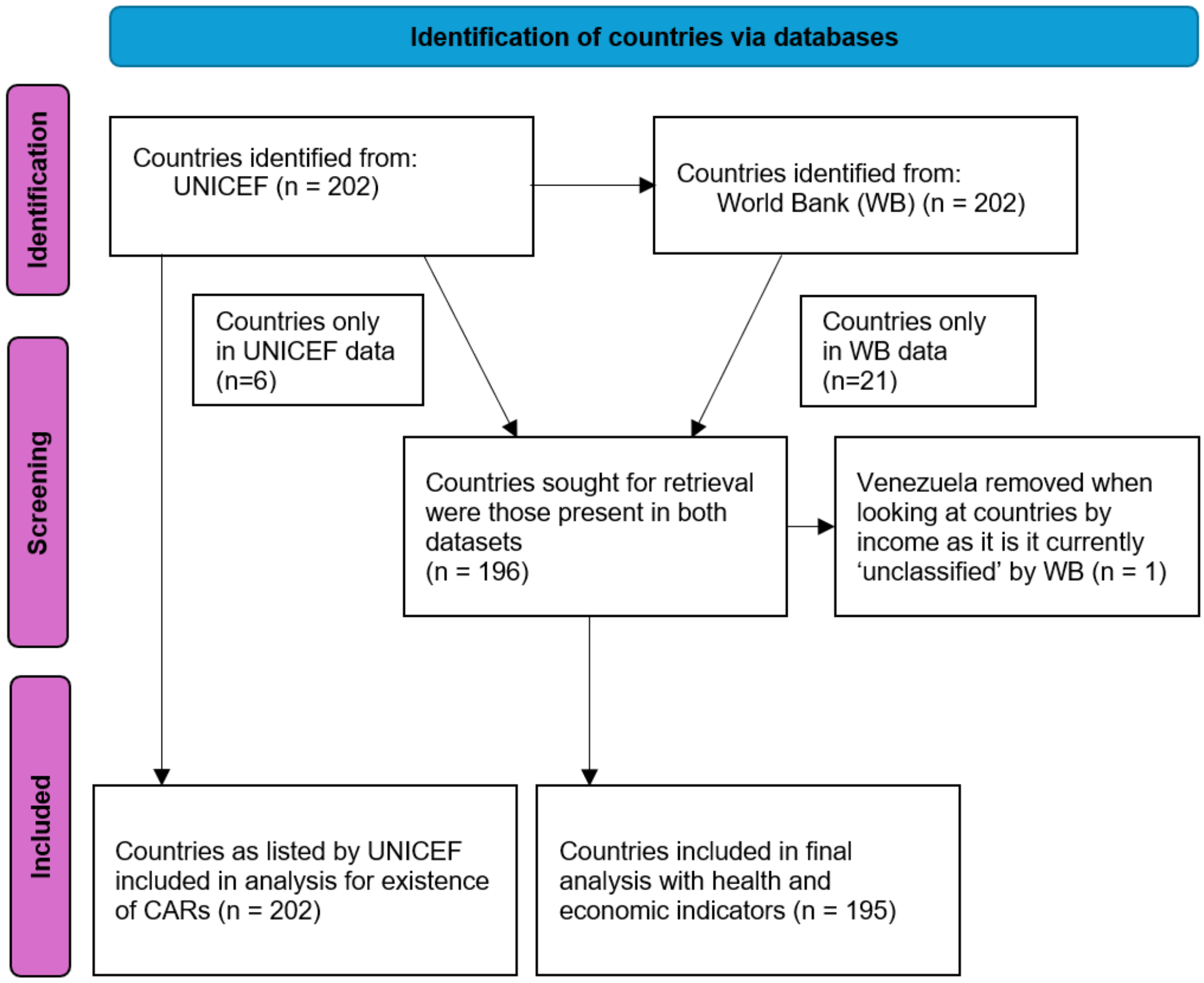

Supplement: Supplementary file 1 [file jogh-16-04263-s001.pdf]
